# Diagnostic values of Xpert MTB/RIF, T-SPOT.TB and adenosine deaminase for HIV-negative tuberculous pericarditis in a high burden setting: a prospective observational study

**DOI:** 10.1038/s41598-020-73220-y

**Published:** 2020-10-01

**Authors:** Xu Hu, Baochun Xing, Wei Wang, Pengwei Yang, Yumei Sun, Xiangyang Zheng, Yaomin Shang, Feifei Chen, Nai Liu, Lu Yang, Yue Zhao, Jiao Tan, Xueya Zhang, Yan Wang, Zhengxun Zhang, Yaqian Liu

**Affiliations:** 1 Department of Tuberculosis, Henan Chest Hospital, Zhengzhou, Henan Province People’s Republic of China; 2 Department of Laboratory Medicine, Henan Chest Hospital, Zhengzhou, Henan Province People’s Republic of China; 3 Department of Cardiology, Henan Chest Hospital, Zhengzhou, Henan Province People’s Republic of China; 4 Department of Cardiac Surgery, Henan Chest Hospital, Zhengzhou, Henan Province People’s Republic of China; 5 Department of Medical Records, Henan Chest Hospital, Zhengzhou, Henan Province People’s Republic of China

**Keywords:** Infectious-disease diagnostics, Tuberculosis

## Abstract

The diagnosis of tuberculous pericarditis (TBP) remains challenging. This prospective study evaluated the diagnostic value of Xpert MTB/RIF (Xpert) and T-SPOT.TB and adenosine deaminase (ADA) for TBP in a high burden setting. A total of 123 HIV-negative patients with suspected TBP were enrolled at a tertiary referral hospital in China. Pericardial fluids were collected and subjected to the three rapid tests, and the results were compared with the final confirmed diagnosis. Of 105 patients in the final analysis, 39 (37.1%) were microbiologically, histopathologically or clinically diagnosed with TBP. The sensitivity, specificity, positive predictive value, negative predictive value, positive likelihood ratio, negative likelihood ratio and diagnostic odds ratio (DOR) for Xpert were 66.7%, 98.5%, 96.3%, 83.3%, 44.0, 0.338, and 130.0, respectively, compared to 92.3%, 87.9%, 81.8%, 95.1%, 7.6, 0.088, and 87.0, respectively, for T-SPOT.TB, and 82.1%, 92.4%, 86.5%, 89.7%, 10.8, 0.194, and 55.8, respectively, for ADA (≥ 40 U/L). ROC curve analysis revealed a cut-off point of 48.5 spot-forming cells per million pericardial effusion mononuclear cells for T-SPOT.TB, which had a DOR value of 183.8, while a cut-off point of 41.5 U/L for ADA had a DOR value of 70.9. Xpert (Step 1: rule-in) followed by T-SPOT.TB [cut-off point] (Step 2: rule-out) showed the highest DOR value of 252.0, with only 5.7% (6/105) of patients misdiagnosed. The two-step algorithm consisting of Xpert and T-SPOT.TB could offer rapid and accurate diagnosis of TBP.

## Introduction

Tuberculosis (TB), which is caused by *Mycobacterium tuberculosis* (MTB) infection, remains the top infectious disease worldwide. China is a high TB burden country that accounts for 9% of global total TB patients^1^. A recent study showed that one-third of HIV-negative TB patients had extrapulmonary TB in China, and extrapulmonary TB patients tended to have diagnostic delays, misdiagnosis and an increased trend of drug-resistant TB^2^.

Tuberculous pericarditis (TBP) is a severe extrapulmonary TB and is the most common reason for pericarditis in high TB burden settings^3^^.^ It is known to increase the risk of poor outcomes, including cardiac tamponade, constrictive pericarditis, and high mortality. Pericardiocentesis remains an essential part of the diagnostic approaches^4,5^. However, the time-consuming MTB culture from pericardial fluids (PF) has poor sensitivity, and invasive pericardial biopsy is often unavailable. Thus, the diagnosis of TBP remains problematic and is often delayed^5,6^. Rapid initiation of anti-TB treatment could reduce the mortality of TBP^7^, and the investigation of rapid and accurate diagnostic tests is essential. Currently, the most widely used rapid biomarkers or assays are adenosine deaminase (ADA) and T-SPOT.TB and Xpert MTB/RIF (Xpert).

ADA is an enzyme in lymphocytes and myeloid cells that is required for the conversion of adenosine to inosine and is essential for DNA metabolism and cell viability. Among lymphocyte-predominant effusions, levels of ADA are typically higher in diseases caused by TB than those caused by other conditions^8^. A previous meta-analysis showed that the pooled sensitivity and specificity of ADA for TBP diagnosis were 90% and 86%, respectively^9^. Probable TBP was commonly defined as the presence of a lymphocytic PF with elevated ADA ≥ 40 U/L^10^, but the optimal cut-off value of ADA levels is still controversial.

T-SPOT.TB is an enzyme-linked immunospot assay, an in vitro test that measures interferon-gamma (IFN-γ) release of activated MTB-specific effector T cells isolated from patients’ blood or serous effusions within 2 days and is not cross-reactive with Bacillus Calmette-Guerin or most non-tuberculous mycobacteria^11^. Because of the migration of effector T cells from peripheral blood to the serous cavity, the diagnostic value of T-SPOT.TB on serous effusions was higher than that on peripheral blood, with a reported sensitivity and specificity of 92.0% and 85.0%, respectively^12^. However, published studies using T-SPOT.TB on PF for TBP diagnosis is limited^13–15^, and the optimal cut-off value of spot-forming cells (SFCs) in pericardial effusion mononuclear cells (PEMC) for T-SPOT.TB is not well studied. Of note, HIV co-infection was associated with the false-negative results of T-SPOT.TB, yielding the lower sensitivity and the higher specificity of T-SPOT.TB in diagnosing HIV-positive TB patients^16,17^. In addition, more indeterminate T-SPOT.TB results were also found in HIV-positive patients^18^.

Xpert is a new quantitative polymerase chain reaction test that has been introduced for the rapid diagnosis of MTB infection and rifampicin (RIF) resistance within 2 h and is endorsed by the World Health Organization (WHO)^19^. A recent diagnostic evaluation study showed that the pooled sensitivity and specificity of Xpert in diagnosing extrapulmonary TB were 75.0% and 98.0%, respectively^20^. However, the diagnostic value of Xpert for TBP diagnosis has not been well studied due to limited data^21–23^. Limitations of the few published works included the relatively small number of TBP patients (usually quoted as a small part of extrapulmonary TB), the unsatisfactory reference standard, and the high proportion of HIV-positive participants in the cohorts.

To address these gaps, we prospectively evaluated the diagnostic value of Xpert and T-SPOT.TB and ADA for the diagnosis of HIV-negative TBP when compared to the composite reference standard (CRS) and determined an optimal algorithm for rapid and accurate diagnosis.

## Materials and methods

The Ethics Committee of Henan Chest Hospital approved the study, and all the experimental protocols of this study were in accordance with the guidelines of the Declaration of Helsinki. Informed consent was obtained from each patient prior to enrolment.

### Patient population

Between February 2018 and January 2019, consecutive patients with suspected TBP were admitted at Henan Chest Hospital in the TB Diagnosis and Treatment Centre of Henan Province in China. Inclusion criteria included the presence of large pericardial effusions amenable to safe pericardiocentesis (greater than 10 mm echo-free space around the heart in diastole), age 18 years or older, corresponding symptoms, and TB contact history or other sites of TB. Exclusion criteria included pregnancy, anti-TB treatment initiation > 1 week prior to pericardiocentesis, and refusal or inability to sign consent. All patients were screened for HIV, and HIV-positive patients were referred to other hospitals that specialize in HIV treatment. Enrolled patients underwent at least 9 months of follow-up to assess the response to treatment.

### Diagnostic sample collection and handling

A minimum of 60 ml PF was collected through percutaneous pericardiocentesis. Pericardial biopsy was performed at the discretion of physicians according to clinical practice. PF samples were subjected to routine biochemical and cytological analysis, measurement of ADA levels (DENUO, Shanghai, China), concentrated fluorescence smear microscopy (Gram stain, acid-fast bacilli stain), liquid culture for MTB using MGIT 960 (BD Diagnostics, Hunt Valley, MD, USA), and a T-SPOT.TB test (Oxford Immunotec, Abingdon, UK) and Xpert test (Cepheid, Sunnyvale, CA, USA). Drug susceptibility testing (DST) was performed on positive culture isolates with the proportion method using Lowenstein-Jensen medium. A critical concentration of 40 mg/mL was used for RIF. All the laboratory staff performing the requested tests were blinded to all microbiological and clinical information.

### ADA assay

An ADA assay was performed on 1–8 ml PF samples collected from each patient according to the manufacturer’s specifications^21^. Samples were either processed immediately or stored (2–4 °C) for processing within 24 h. The Diazyme ADA assay is based on the enzymatic deamination of adenosine to inosine, which is converted to hypoxanthine by purine nucleoside phosphorylase. The reagent was used at 37 °C on an instrument that is capable of reading absorbance accurately at 560 nm. ADA activity was measured as units per litre (U/L), where one unit of ADA is defined as the amount of ADA that generates 1 μmol of inosine from adenosine per minute at 37 °C.

### T-SPOT.TB assay

A 50 ml PF sample was collected from each patient and analysed within 6 h after collection according to the manufacturer’s specifications. T-SPOT.TB utilized AIM-V as a negative control, PHA as a positive control, and ESAT-6 and CFP-10 as specific antigens. PEMCs were separated by Ficoll-Hypaque gradient centrifugation and plated (2.5 × 10^5^ per well) on a plate precoated with an antibody against IFN-γ. After incubation for 16–18 h at 37 °C in 5% carbon dioxide, plate wells were washed and incubated with a conjugate against the antibody used and an enzyme substrate. SFCs that represented antigen-specific T cells secreting IFN-γ were counted with an automated ELISPOT reader (AID-ispot, Strassberg, Germany). The positive response was defined as 6 or more spots and twice the number of spots compared with the negative control well. The background number of spots in the negative control well should be less than 20 spots^12,13^.

### Xpert MTB/RIF assay

The Xpert assay was performed on PF samples using the manufacturer’s specifications for sputum samples as previously described^19^. Xpert was performed using 1 ml of unconcentrated and unprocessed PF. The PF and sample buffer solutions were mixed vigorously and incubated at room temperature for 15 min, with further mixing halfway through the incubation. A 2 ml volume of the diluted sample was then transferred to an Xpert cartridge and was arranged in the detection module to start automatic detection. All Xpert results were available within 2 h, and the experiment was repeated twice.

### Diagnostic classification

The diagnosis of TBP was determined by physicians independent of ADA and T-SPOT.TB and Xpert results. Both of the following conditions could be diagnosed as TB: (1) identification of MTB in PF by acid-fast bacilli stain and culture, or by the presence of granulomas in pericardial tissue (definite TB); (2) clinical and radiological evidence of TBP, lymphocytic predominance in PF, and positive response to anti-TB treatment (clinical TB). The following conditions could be diagnosed as non-TB: an available alternative diagnosis was given or improvement without anti-TB treatment.

### Statistical analysis

The Statistical Package for the Social Sciences (SPSS) (Version 22.0, IBM Corp., Armonk, NY, USA) was utilized for statistical analysis. Sensitivity, specificity, positive predictive value (PPV), negative predictive value (NPV), positive likelihood ratio (PLR), negative likelihood ratio (NLR) and diagnostic odds ratio (DOR) were calculated to evaluate the diagnostic value of ADA and T-SPOT.TB, and Xpert tests. The area under the receiver operating characteristic curve (AUC) of the T-SPOT.TB and ADA tests of the diagnostic cut-off values were calculated. DOR was calculated as the ratio of the odds of positivity in a disease condition relative to the odds of positivity in the non-disease condition, with higher values indicating better diagnostic discriminatory test performance^24^. The difference in means was assessed using Student’s t test or the Mann–Whitney U test. The proportions were compared using the χ^2^ or Fisher’s exact test as appropriate. Ninety-five percent confidence intervals (95% CI) were estimated according to the binomial distribution. The threshold for significance was *P* < 0.05.

## Results

### Clinical characteristics

**Figure 1:**
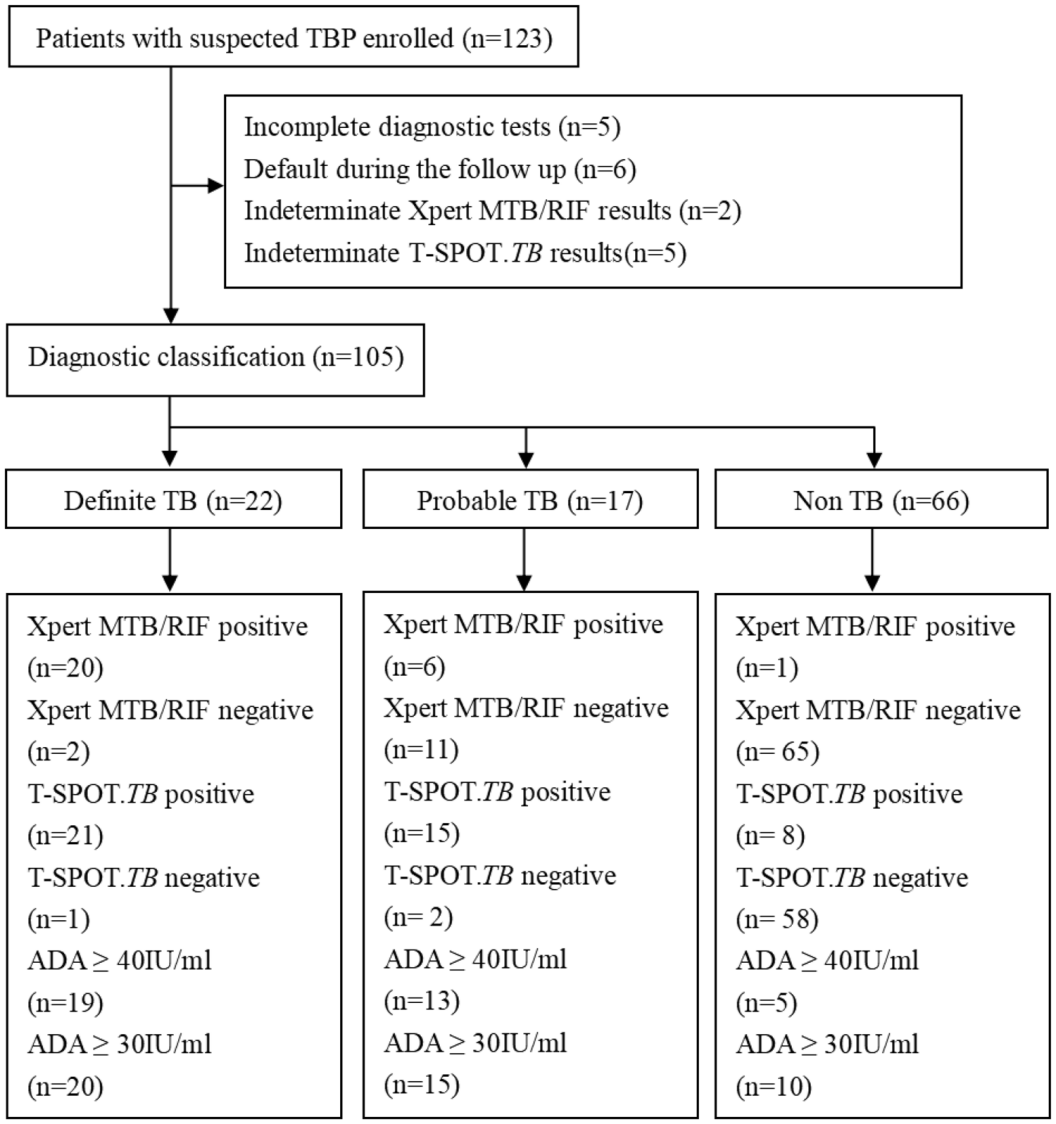
Flowchart showing the classification and the test results of patients enrolled in the study. Abbreviations: TBP, Tuberculous pericarditis; TB, Tuberculosis; ADA, adenosine deaminase.

A total of 123 suspected TBP patients were prospectively enrolled. Eighteen patients were excluded due to incomplete diagnostic tests (n = 5), default during the follow-up (n = 6), or indeterminate Xpert or T-SPOT.TB results (n = 7). Of the 105 patients in the final analysis, 39 (37.1%) were diagnosed with TBP according to the CRS, of which 20, 2, and 17 patients were microbiologically, histopathologically and clinically diagnosed, respectively, and 66 (62.9%) were diagnosed with non-TB. The flow chart is shown in Fig. 1.

**Figure 2:**
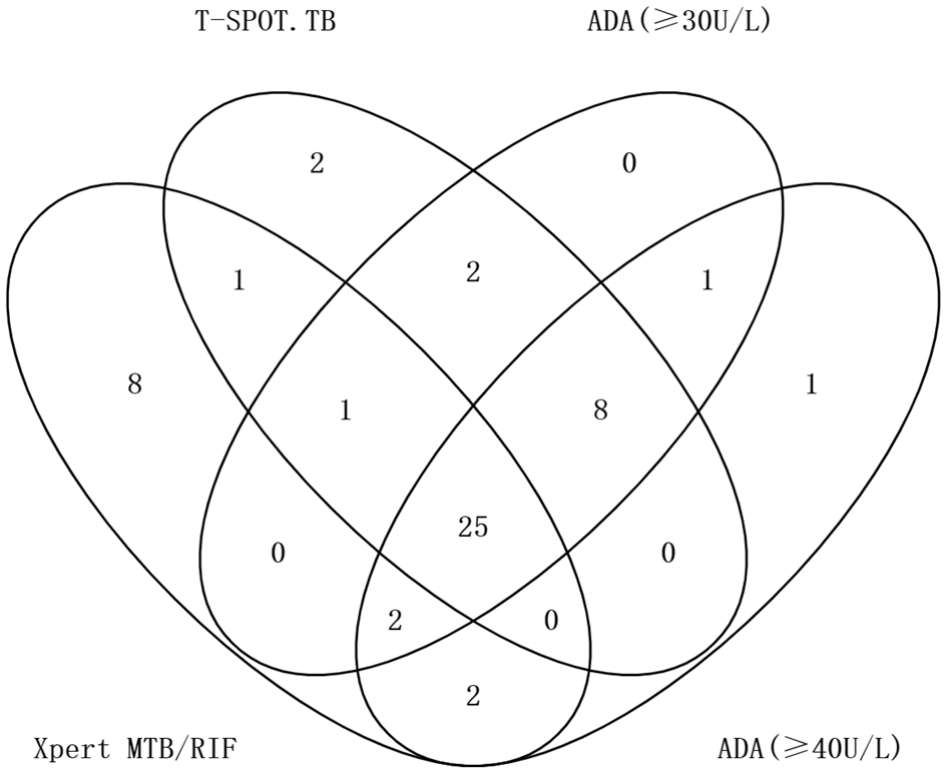
Venn diagram of the overlap among different diagnostics for pericardial fluid testing. Abbreviations: ADA, adenosine deaminase.

Among the 39 TBP patients, 26 were detected by Xpert, compared to 36, 35, and 32 by T-SPOT.TB, ADA (≥ 30 U/L) and ADA (≥ 40 U/L), respectively (Fig. 2). Among the 66 non-TB patients, 29 were diagnosed with solid and haematological malignant tumours, 9 were diagnosed with heart failure, 6 were diagnosed with renal failure, 12 were diagnosed with connective tissue diseases, and 10 were diagnosed with idiopathic pericarditis. Bacterial and fungal cultures were negative for all patients.

TBP patients were younger than non-TB patients (*P* = 0.017). A high proportion of TBP patients (20.5%) had other sites involved in TB compared with the non-TB patients (4.5%, *P* = 0.024). The clinical features are shown in Table 1.

**Table 1:** Clinical features of patients with suspected tuberculous pericarditis.

Baseline characteristics	All (n = 105)	TB (n = 39)	Non-TB (n = 66)	*P* value
Age (years, median, IQR)	48.0 (42.0–62.5)	46.0 (42.0–51.0)	53.0 (42.0–64.0)	0.017
Male (n, %)	56 (53.3)	21 (53.8)	35 (53.0)	0.935
NYHA Class I–II (n, %)	76 (72.4)	30 (76.9)	46 (69.7)	0.424
Systolic blood pressure (mmHg, median, IQR)	120.0 (107.5–130.0)	115.0 (105.0–125.0)	120.0 (115.0–131.3)	0.027
Diastolic blood pressure (mmHg, median, IQR)	75.0 (67.5–85.0)	70.0 (65.0–85.0)	75.0 (73.8–85.0)	0.029
Heart rate (median, IQR)	106.0 (96.0–117.5)	106.0 (96.0–119.0)	106.0 (96.0–117.3)	0.958
Pericardial tamponade (n, %)	13 (12.4)	3 (7.7)	10 (15.2)	0.415
Other sites involved of TB (n, %)	11 (10.5)	8 (20.5)	3 (4.5)	0.024
Serum biochemical data				
Total leukocyte (*109/L, median, IQR)	7.3 (5.5–8.8)	7.3 (5.7–8.8)	7.3 (5.2–8.7)	0.492
ESR (mm/h, median, IQR)	34.0 (22.0–51.0)	45.0 (29.0–67.0)	26.0 (15.0–36.3)	0.000
CRP (mg/L, median, IQR)	9.8 (5.1–18.8)	16.7 (12.5–25.3)	6.1 (3.2–11.2)	0.000
Routine PF analysis				
Total protein (g/L, median, IQR)	35.9 (28.7– 44.2)	41.6 (35.4–55.2)	33.3 (23.8–39.9)	0.000
Lactate dehydrogenase (U/L, median, IQR)	487.5 (389.5–653.7)	450.2 (389.5–640.5)	615.0 (383.3–718.3)	0.138
Glucose (mmol/L, median, IQR)	16.4 (12.9–18.2)	15.4 (11.8–17.3)	16.9 (13.2–18.5)	0.143
ADA (U/L, median, IQR)	22.0 (19.0–62.0)	74.0 (43.0–82.0)	20.0 (15.0–22.5)	0.000

TB, tuberculosis; NYHA, New York Heart Association; IQR, interquartile range; PF, pericardial fluid; ESR, erythrocyte sedimentation rate; CRP, c-reactive protein; ADA, adenosine deaminase.

### Diagnostic values of ADA

**Figure 3:**
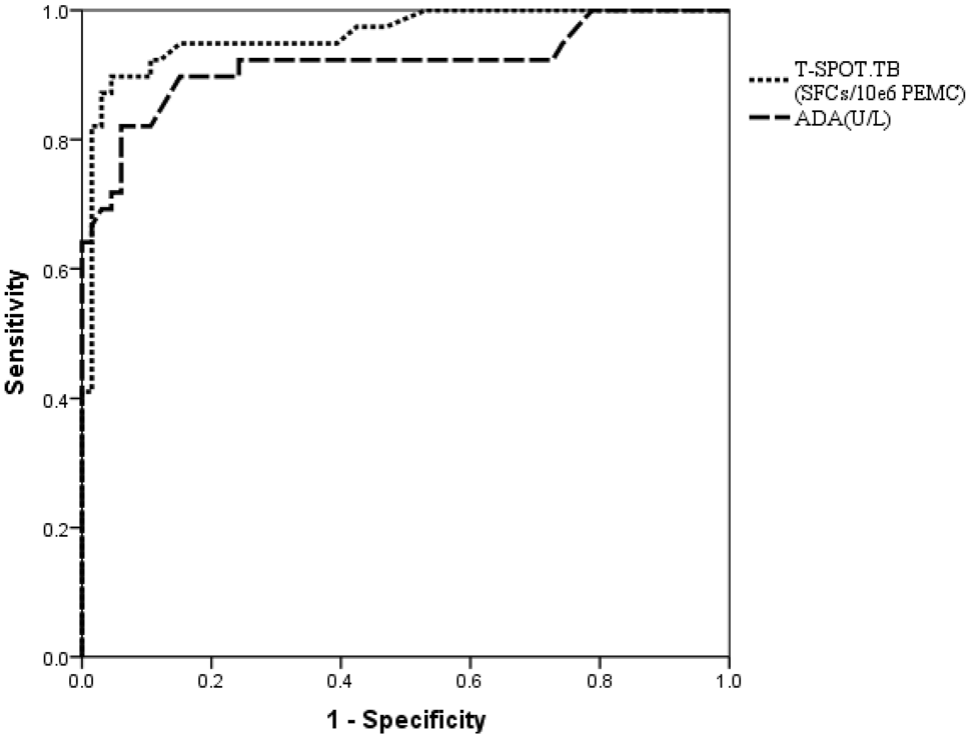
Comparison of receiver operator characteristics curves for T-SPOT.TB and ADA. Abbreviations: ADA, adenosine deaminase.

In TBP patients, the median (IQR) level of ADA was 74.0 (43.0–82.0) U/L, which was significantly higher than in non-TB effusions (20[15–22.5], *P* < 0.001). Using a clinical cut-off point of 30 U/L, evaluation of the efficiency of ADA showed that it had sensitivity, specificity, PPV, NPV, PLR, NLR, and DOR values of 89.7%, 84.8%, 77.8%, 93.3%, 5.9, 0.121, and 49.0, respectively (Table 2). When using a clinical cut-off point of 40 U/L, it had sensitivity, specificity, PPV, NPV, PLR, NLR, and DOR values of 82.1%, 92.4%, 86.5%, 89.7%, 10.8, 0.194, and 55.8, respectively (Table 2). Based on the ROC curve analysis, the cut-off value of ADA was 41.5 U/L, showing sensitivity, specificity, PPV, NPV, PLR, NLR, and DOR values of 82.1%, 93.9%, 88.9%, 89.9%, 13.5, 0.191, and 70.9, respectively (Table 2). The AUC for ADA was 0.917 (Fig. 3).

**Table 2:** Diagnostic performance of Xpert MTB/RIF, T-SPOT.TB and ADA.

Diagnostic tests		Values (95%CI, n/N)				Ratio (95%CI)		
		Sensitivity%	Specificity%	PPV%	NPV%	PLR	NLR	DOR
Single test	Xpert MTB/RIF	66.7%^abcdefgh^ (49.7–80.4), 26/39	98.5%^ijlm^ (90.7–99.9), 65/66	96.3%^qr^ (79.1–99.8), 26/27	83.3%^stuvw^ (72.8–90.5), 65/78	44.0 (6.2–311.6)	0.338 (0.217–0.528)	130.0 (16.2–1044.9)
	T-SPOT.TB (cut-point in current clinical use: 6 or more spots and had twice the number of spots than the negative control well)	92.3%^a^ (78.0–98.0), 36/39	87.9%^i^ (77.0–94.3), 58/66	81.8% (66.8–91.3), 36/44	95.1%^s^ (85.4–98.7), 58/61	7.6 (4.0–14.7)	0.088 (0.029–0.261)	87.0 (21.7–349.5)
	T-SPOT.TB (ROC-selected cut-off value: ≥ 48.5 SFCs /10^6^ PEMC)	89.7%^b^ (74.8–96.7), 35/39	95.5%^kn^ (86.4–98.8), 63/66	92.1% (77.5–97.9), 35/38	94.0%^t^ (84.7–98.1), 63/67	19.7 (6.5–59.9)	0.107 (0.042–0.272)	183.8 (38.9–868.3)
	ADA (cut-point in current clinical use: ≥ 30 U/L)	89.7%^c^ (74.8–96.7), 35/39	84.8%^jko^ (73.4–92.1), 56/66	77.8%^q^ (62.5–88.3), 35/45	93.3% (83.0–97.8), 56/60	5.9 (3.3–10.6)	0.121 (0.048–0.307)	49.0 (14.3–168.3)
	ADA (cut-point in current clinical use: ≥ 40 U/L)	82.1% (65.9–91.9), 32/39	92.4% (82.5–97.2), 61/66	86.5% (70.4–94.9), 32/37	89.7% (79.3–95.4), 61/68	10.8 (4.6–25.5)	0.194 (0.099–0.381)	55.8 (16.4–189.8)
	ADA (ROC-selected cut-off value : ≥ 41.5 U/L)	82.1% (65.9–91.9), 32/39	93.9% (84.4–98.0), 62/66	88.9% (73.0–96.4), 32/36	89.9% (79.6–95.5), 62/69	13.5 (5.2–35.4)	0.191 (0.097–0.374)	70.9 (19.3–260.1)
Two-step algorithm	Xpert MTB combined with T-SPOT.TB (with T-SPOT.TB if Xpert MTB/RIF negative)	94.9%^d^ (81.4–99.1), 37/39	87.9%^l^ (77.0–94.3), 58/66	82.2% (67.4–91.5), 37/45	96.7%^u^ (87.5–99.4), 58/60	7.8 (4.1–15.0)	0.058 (0.015–0.226)	134.1 (27.0–666.6)
	Xpert MTB combined with T-SPOT.TB(≥ 48.5 SFCs /10^6^ PEMC) (with T-SPOT.TB if Xpert MTB/RIF negative)	92.3%^e^ (78.0–98.0), 36/39	95.5%^op^ (86.4–98.8), 63/66	92.3% (78.0–98.0), 36/39	95.5%^v^ (86.4–98.8), 63/66	20.3 (6.7–61.6)	0.081 (0.027–0.239)	252.0 (48.3–1314.6)
	Xpert MTB combined with ADA(≥ 30 U/L) (with ADA if Xpert MTB/RIF negative)	92.3%^f^ (78.0–98.0), 36/39	84.8%^mnp^ (73.4–92.1), 56/66	78.3%^r^ (63.2–88.5), 36/46	94.9%^w^ (84.9–98.7), 56/59	6.1 (3.4–10.9)	0.091 (0.03–0.27)	67.2 (17.3–260.9)
	Xpert MTB combined with ADA(≥ 40 U/L) (with ADA if Xpert MTB/RIF negative)	87.2%^g^ (71.8–95.2), 34/39	92.4% (82.5–97.2), 61/66	87.2% (71.8–95.2), 34/39	92.4% (82.5–97.2), 61/66	11.5 (4.9–27.0)	0.138 (0.061–0.315)	83.0 (22.4–307.0)
	Xpert MTB combined with ADA(≥ 41.5U/L) (with ADA if Xpert MTB/RIF negative)	87.2%^h^ (71.8–95.2), 34/39	93.9% (84.4–98.0), 62/66	89.5% (74.3–96.6), 34/38	92.5% (82.7–97.2), 62/67	14.4 (5.5–37.5)	0.136 (0.06–0.31)	105.4 (26.5–418.8)

PPV, positive predictive value; NPV, negative predictive value; PLR, positive likelihood ratio; NLR, negative likelihood ratio; DOR, diagnostic odds ratio.

Letters a, b, c, d, e, f, g, h, i, j, k, l, m, n, o, and p were used to indicate which groups were being compared for statistical analysis: a, e, f, j, m: *P* = 0.005; b, c: *P* = 0.014; d: *P* = 0.002; g, h: *P* = 0.032; i, l: *P* = 0.016; k, n, o, p: *P* = 0.041; q: *P* = 0.044; r: *P* = 0.046; s: *P* = 0.031; t: *P* = 0.046; u: *P* = 0.013; v: *P* = 0.021; w: *P* = 0.037.

### Diagnostic values of T-SPOT.TB

In TBP patients, the frequency of SFCs was 229 per million PEMC (IQR, 86–492), which was significantly higher than in non-TB patients (14 ^7–23^, *P* < 0.001). Evaluation of the efficiency of the T-SPOT.TB assay (cut-off point in current clinical use: 6 or more spots and twice the number of spots than the negative control well) showed that it had sensitivity, specificity, PPV, NPV, PLR, NLR, and DOR values of 92.3%, 87.9%, 81.8%, 95.1%, 7.6, 0.088, and 87.0, respectively (Table 2). Based on the ROC curve analysis, the cut-off value was 48.5 SFCs/10^6^ PEMC. Evaluation of the efficiency of the T-SPOT.TB assay (ROC-selected cut-off value: ≥ 48.5 SFCs/10^6^ PEMC) showed that it had sensitivity, specificity, PPV, NPV, PLR, NLR, and DOR values of 89.7%, 95.5%, 92.1%, 94.0%, 19.7, 0.107, and 183.8, respectively (Table 2). The AUC for T-SPOT.TB was 0.962 (Fig. 3).

### Diagnostic values of Xpert MTB/RIF

Evaluation of the efficiency of the Xpert assay showed that it had sensitivity, specificity, PPV, NPV, PLR, NLR, and DOR values of 66.7%, 98.5%, 96.3%, 83.3%, 44.0, 0.338, and 130.0, respectively (Table 2). Among the 20 TBP cases with positive MTB culture, Xpert had 4 RIF-resistant cases and 16 RIF-sensitive cases. Comparison among 14 cases with available DST outcomes and eligible Xpert results showed that Xpert correctly identified all 3 RIF-resistant cases and 11 RIF-sensitive cases defined by DST. In addition, among 19 TBP cases with negative MTB culture, Xpert showed 8 RIF-resistant cases.

### Comparison of diagnostic accuracy among three rapid tests

Table 2 compares the diagnostic accuracy of the three rapid diagnostic tests. The sensitivity from highest to lowest was T-SPOT.TB, ADA (≥ 30 U/L)/T-SPOT.TB (≥ 48.5 SFCs/10^6^ PEMC [cut-off value]), ADA (≥ 40 U/L)/ADA (≥ 41.5 U/L [cut-off value]), and Xpert. The specificity from highest to lowest was Xpert, T-SPOT.TB (≥ 48.5 SFCs/10^6^ PEMC), ADA (≥ 41.5 U/L), ADA (≥ 40 U/L), T-SPOT.TB and ADA (≥ 30 U/L). The DORs from highest to lowest were T-SPOT.TB (≥ 48.5 SFCs/10^6^ PEMC), Xpert, T-SPOT.TB, ADA (≥ 41.5 U/L), ADA (≥ 40 U/L) and ADA (≥ 30 U/L).

The sensitivity and NPV of Xpert were lower than those of T-SPOT.TB, T-SPOT.TB (≥ 48.5 SFCs/10^6^ PEMC), and ADA (≥ 30 U/L) (*P* < 0.05), while the specificity of Xpert was higher than that of T-SPOT.TB and ADA (≥ 30 U/L) (*P* < 0.05), and the PPV of Xpert was higher than that of ADA (≥ 30 U/L) (*P* < 0.05). The specificity of T-SPOT.TB (≥ 48.5 SFCs/10^6^ PEMC) was higher than that of ADA (≥ 30 U/L) (*P* < 0.05).

### Diagnostic values of the two-step algorithm

**Figure 4:**
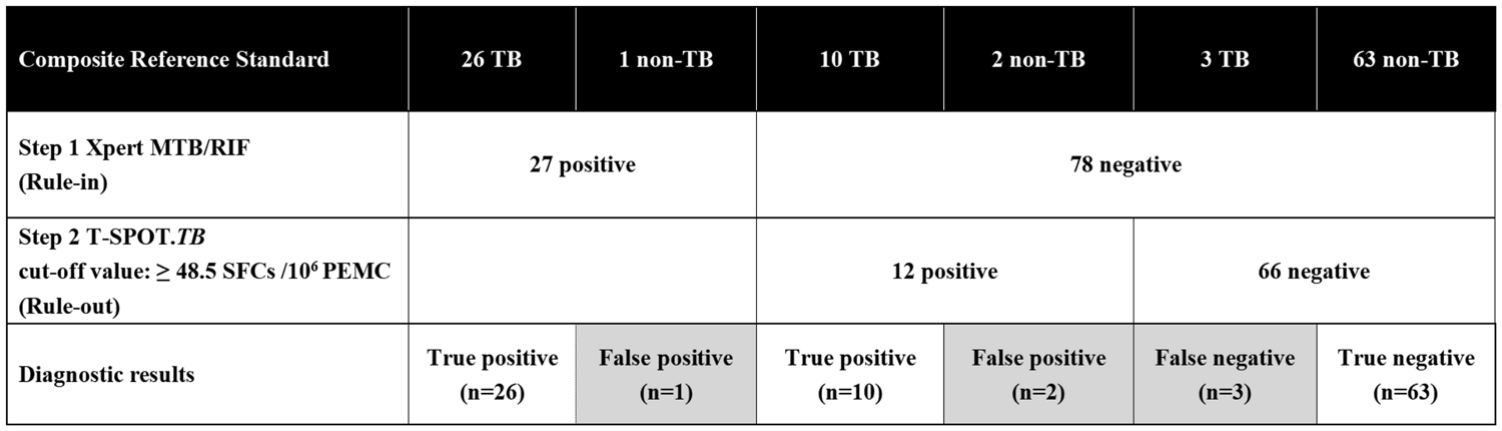
A two-step algorithm using Xpert followed by T-SPOT.TB could be used for the rapid and accurate diagnosis of TBP. Using Xpert as Step 1(rule-in), 27 of 105 applicants were Xpert-positive and 78 applicants were Xpert-negative. Then using T-SPOT.TB (≥ 48.5 SFCs/10^6^ PEMC[cut-off value]) as Step 2(rule-out), 12 of 78 applicants were T-SPOT-positive and 66 applicants were T-SPOT-negative. Overall, only 5.7%(6/105) of patients were misdiagnosed. Black: individuals defined with composite reference standard; Gray: individuals with false results for the indicated test.

Table 2 shows the diagnostic accuracy of Xpert combined with T-SPOT.TB or ADA. Performing Xpert (step 1: rule-in) followed by either T-SPOT.TB or ADA (step 2: rule-out) offered superior diagnostic accuracy when compared to T-SPOT.TB or ADA alone. The two-step algorithm consisting of Xpert and T-SPOT.TB (≥ 48.5 SFCs/10^6^ PEMC[cut-off value]) showed excellent diagnostic accuracy with a sensitivity of 92.3%, specificity of 95.5%, PPV of 92.3%, NPV of 95.5%, PLR of 20.3, NLR of 0.081, and DOR of 252.0, with only 5.7% (6/105) of patients misdiagnosed (Fig. 4).

## Discussion

Given the lack of rapid and reliable diagnostic tests for TBP, we prospectively evaluated the utility of the Xpert and T-SPOT.TB and ADA assays in HIV-negative cohorts using CRS as reference standard. To the best of our knowledge, this is the first prospective study to comprehensively evaluate the diagnostic value of the above three rapid tests on PF individually and in tandem. In addition, this is the largest study (123 participants) to evaluate the utility of Xpert or T-SPOT.TB on PF in the HIV-negative population. Our key findings were as follows: (1) When compared to those of ADA and T-SPOT.TB, Xpert had the highest specificity, PLR and PPV, making it the rule-in test for TBP. However, Xpert also had a high NPV; thus, the alternative diagnosis confirmed by other diagnostic tests is essential in high burden settings; (2) T-SPOT.TB had the highest sensitivity and NPV and the lowest NLR, making it the rule-out test. According to the DOC curve, 48.5 SFCs per million PEMC was the optimal cut-off value of T-SPOT.TB, which showed the best diagnostic accuracy among the single diagnostic tests (DOR = 183.8); (3) the two clinical cut-off points (30 U/L and 40 U/L) of ADA showed no statistically significant difference in diagnosing TBP, and 41.5 U/L was the optimal cut-off value; (4) The two-step algorithm using Xpert followed by T-SPOT.TB (≥ 48.5 SFCs/10^6^ PEMC [cut-off value]) showed excellent diagnostic accuracy (DOR = 252.0) with a sensitivity and specificity of 92.3% and 95.5%, respectively.

For TBP patients, the presence of HIV infection contributed to the complexity of the disease process considerably^25^. The proportion of microbiologically confirmed TBP patients in HIV-negative cohorts was higher than that in HIV-positive cohorts^26^, and the positive histopathological results of pericardial tissue, such as granuloma, were poor in HIV-positive patients^27^. The sensitivity of Xpert for TBP diagnosis was higher in HIV-positive patients than HIV-negative patients, corresponding to higher bacillary loads in the PF of HIV-positive patients^21^. HIV viral load was higher in PF than in plasma of HIV-positive patients and was inversely correlated with the proportion of CD4+ T cells in PF^28^; thus, HIV co-infection was associated with false-negative results and the lower sensitivity of T-SPOT.TB^16,17^. In addition, more indeterminate T-SPOT.TB results were also found in HIV-positive patients^18^. Most studies suggested that HIV status had no significant impact on the diagnostic value of ADA. However, lower ADA levels were observed in HIV-positive patients with severe CD4 lymphocyte depletion^4^. Due to the complex influence of HIV status on the diagnostic value of different tests and the very low prevalence of HIV positivity among TB patients in China^29^, we excluded HIV-positive patients to reduce heterogeneity.

The proportion of TBP in pericarditis was estimated to be 4% in the Western world and up to 70% in South Africa^3,30^. In our study, 37.1% (39/105) of suspected patients were diagnosed with TBP, which is higher than previous reports from China^13,31^. As the study was conducted in a tertiary referral hospital that was the diagnosis and treatment centre of TB, a high proportion of TB patients might be enrolled.

Imperfect reference standards may lead to the misclassification of cases in diagnostic validity studies. The reported positive rate of conventional MTB culture on PF was only 53%, and the sensitivity of pericardial biopsy varied from 10 to 64%^5^. In addition, pericardial biopsy was invasive, and the histopathological results were influenced by the location of the biopsy site, the quality of equipment, and the experience of physicians^14^. Thus, the number of false-positive cases might be overestimated when diagnostic tests are evaluated against culture or histopathology, leading to underestimation of specificity. In addition, the overestimation of sensitivity could be corrected by bringing clinical data into the reference standard. We believe that using CRS as a reference standard in our study could better reflect the true performance of each diagnostic test.

In our study, the sensitivity, specificity and DOR of Xpert were 66.7%, 98.5% and 130.0, respectively, when compared to CRS, showing the superior diagnostic performance of TBP. Xpert also detected all RIF-resistant cases defined by the time-consuming DST in our study. Another prospective study, which enrolled a high proportion of HIV-positive TBP patients, found that Xpert had a sensitivity and specificity of 63.8% and 100%, respectively, when compared with culture and histopathology^21^. The sensitivity of Xpert was higher in HIV-positive patients due to the higher bacillary loads but was underestimated when compared with culture and histopathology. Thus, the results of that study were similar to ours. TBP is commonly believed to be a paucibacillary condition driven by an intense delayed-type hypersensitivity response to TB antigens^5^, which is consistent with pleural or peritoneal TB. However, the sensitivity (66.7%) of Xpert on PF in our study was higher than those reported in pleural fluids (30%) or peritoneal fluids (32%)^20^. A recent study showed that both HIV-positive and HIV-negative TBP patients confirmed by culture had a high bacillary burden, which could increase mortality^32^. Thus, high or low bacterial burden still remains under debate in TBP patients, and we suggest that Xpert might outperform in TBP than other TB serositis.

One 69-year-old female patient with malignant pericardial tumour and vertebral TB history was misdiagnosed with TBP by Xpert in our study. She was diagnosed with vertebral TB according to CT-guided needle biopsy 4 years ago and was successfully treated after 12 months of anti-tuberculosis treatment. Similar false-positive Xpert results were also reported in a malignant pleural tumour patient^33^ and patients with TB history^34^. Regarding the high TB burden in China, we speculated that this patient might still have latent MTB infection or might have both pericardial tumours and TBP.

The diagnostic values of T-SPOT.TB (DOR 87.0) for TBP in our study were superior to those for pleural TB (DOR 46.99) and TB peritonitis (DOR 26.46)^18^. The diagnostic performance of T-SPOT.TB varied among different sites of extrapulmonary TB^35^, and the utilization of T-SPOT.TB on PF still needs further study. A retrospective study of 75 HIV-negative patients showed the sensitivity and specificity of T-SPOT.TB for TBP diagnosis were both 92% when compared to CRS^13^, in which the sensitivity was similar to ours, while the specificity was higher than ours. As the proportion of definite TB cases was much lower in this retrospective study (3/75), the specificity might be overestimated. Since bacterial replication and immunosuppression were accompanied by increased MTB-specific antigen responses, a cut-off value of frequencies of MTB-specific IFN-γ secreting cells could be established to diagnose active TB^36^. We found the optimal diagnostic cut-off value of T-SPOT.TB was 48.5 SFCs/10^6^ PEMC, which offered the best diagnostic performance among the single diagnostic tests with a sensitivity of 89.7%, a specificity of 95.5% and a DOR 183.8. Another prospective study utilized T-SPOT.TB on serous effusion mononuclear cells (SEMC) for HIV-negative TB serositis, and the results showed that the optimal cut-off value was 56 SFCs/10^6^ SEMC with a sensitivity of 90.5% and a specificity of 89.2%^12^, which is similar to our results.

In our study, 3 TBP patients with low serum lymphocytes gave negative T-SPOT.TB results, in whom 2 were diagnosed as disseminated TB. The sensitivity of T-SPOT.TB would decrease with a lower lymphocyte count^12^, and false-negative results were more common in samples from patients with acute extrapulmonary TB^35^. In addition, a weak MTB-specific IFN-γ response could occur in active TB patients due to immune anergy^12^. A previous study also found TB patients who had false-negative T-SPOT.TB results might be sicker and were associated with increased risk for death^16^. Eight non-TB patients had positive T-SPOT.TB results, 6 of whom had pericardial malignant tumours and 2 had systemic lupus erythematosus, which indicated that aberrant immune activation might influence the diagnostic accuracy of T-SPOT.TB^12^. The latest mathematical model analysis results show that the global burden of latent TB infection (LTBI) was 23.0%, amounting to approximately 1.7 billion people^37^. Patients with LTBI might also show positive results in countries with a high TB burden, such as China^35,38^, which could explain the relatively low PPV (81.8%) of the T-SPOT.TB test in our study.

ADA remains the most widely used biochemical test for diagnosing TBP, and the most common threshold is greater than 40 U/L, showing a sensitivity of 87–93% and specificity of 89–97%^6,10^. Along with the lower threshold of greater than 30 U/L, the sensitivity is higher (94%), but the specificity is lower (68%)^39^. We also found similar results. We found that the cut-off value of ADA was 41.5 U/L, which had sensitivity, specificity, and DOR values of 82.1%, 93.9%, and 70.9, respectively. A previous meta-analysis also showed that the pooled sensitivity, specificity, and DOR of ADA for TBP diagnosis were 90.0%, 86.0% and 42.55, respectively, when using cut-off values ranging from 32.5 to 72 U/L^9^. Although ADA has a number of advantages, including acceptable diagnostic performance, easier accessibility and lower costs, the variable cut-off values still make it difficult to apply in clinical practice. Thus, we suggest that the diagnostic results of ADA should be analysed together with other tests.

In this study, we compared the utility of Xpert, T-SPOT.TB and ADA, focusing on the diagnostic priorities of rapid rule-in and rule-out. We found that performing the T-SPOT.TB test (≥ 48.5 SFCs/10^6^ PEMC [cut-off value]) in Xpert-negative patients could maximize the diagnostic accuracy (DOR = 285.0) with a sensitivity of 92.3% and a specificity of 95.5%. As a result, only 5.7% (6/105) of suspected TBP patients were misdiagnosed. Another prospective study also showed that Xpert combined with either ADA or unstimulated IFN-γ could offer > 97% sensitivity and specificity for TBP diagnosis^21^. However, the time taken for the T-SPOT.TB (48 h) was longer than ADA, and the application of T-SPOT.TB required a more sophisticated laboratory. Furthermore, the cost-effectiveness of the two-step algorithm needs further consideration in resource-limited conditions.

Our study had several limitations. First, only 2 patients in our study were diagnosed by histopathology because pericardial biopsy was not routinely performed in most TB endemic settings. Using pericardial tissue might increase the sensitivity of Xpert^14^, but further studies are needed. Second, Xpert Ultra, the next-generation Xpert that has better capabilities for TB detection, was not evaluated in our study but had been shown to produce higher sensitivity than Xpert on pleural fluids^36^. Thus, the utility of Xpert Ultra may be more promising for TBP diagnosis. Third, the ADA isoenzyme had better diagnostic performance than ADA alone, and ADA2 might be a useful measure of mycobacterial load in patients with pleural effusions^40^. However, the commercial ADA Assay Kit in our laboratory could not evaluate ADA2 activities, and we will evaluate the ADA2 activities in future studies to strengthen our results. Fourth, we excluded indeterminate T-SPOT.TB or Xpert results from the final analysis. Bringing the indeterminate results into a false-negative group might affect the real clinical decision-making scenario^41^, but it still has selection bias. Nonetheless, we believe that the main findings of our study could be extrapolated despite the above limitations.

## Conclusions

For the diagnosis of TBP, Xpert and T-SPOT.TB both showed good diagnostic performance and could be offered as rapid rule-in and rule-out tests, respectively. The two-step algorithm consisting of Xpert and T-SPOT.TB could offer rapid and accurate diagnosis of HIV-negative TBP in high burden settings, especially where DST is desirable.
